# Trend analysis of pediatric urolithiasis prevalence from 1990 to 2021 in the BRICS

**DOI:** 10.3389/fped.2025.1551046

**Published:** 2025-02-21

**Authors:** Zhi-gang Zhang, Qing-cheng Lin, Qing-ying Zhou, Nai-fen Xu, Ding-qin Zheng, Qi-zhuang Pan, Xin-jun Wang, Ran Xu

**Affiliations:** ^1^ Department of Urology, Pingyang Hospital of Wenzhou Medical University, Wenzhou, China; ^2^ Department of Xiaojiang, Pingyang Hospital of Wenzhou Medical University, Wenzhou, China; ^3^ Department of Urology, Zhongshan Hospital Xiamen University, School of Medicine, Xiamen University, Xiamen, China

**Keywords:** urolithiasis, Global Burden of Disease, joinpoint regression, age-period-cohort analysis, prevalence

## Abstract

**Background:**

The increasing epidemiological trend of pediatric urolithiasis over the past three decades has brought it to the forefront of public health attention. An analysis of the disease burden in Brazil, Russia, India, China, and South Africa (BRICS) countries, which share common characteristics such as large population base and limited public health resources, will provide an important reference for global public health policy development. Therefore, this study aimed to investigate the trend of the prevalence of pediatric urolithiasis in BRICS countries during 1990–2021, which in turn will provide more valuable information for them and the world in the prevention and treatment of pediatric urolithiasis.

**Methods:**

In this study, data were obtained from the Global Burden of Disease (GBD) database. The data were then statistically analyzed using the Joinpoint regression model, AutoRegressive Integrated Moving Average (ARIMA) prediction model, and subgroup analysis to assess trends in the prevalence of pediatric urolithiasis.

**Result:**

Globally, the prevalence has been increasing every year, with the greatest increase in the 10–14 age group. Encouragingly, the Age-Standardized Prevalence Rate (ASPR) has shown a decreasing trend. The disease burden of pediatric urolithiasis is higher in India and Russia, with the prevalence in India and ASPR in Russia being the highest in the BRICS countries. In South Africa, there is a clear deficit in prevention and treatment in the 0–4 year age group. Additionally, although the burden of pediatric urolithiasis in Brazil is not currently severe, the trend is the fastest deteriorating among the BRICS countries. Finally, China has made significant progress in the prevention and control of pediatric urolithiasis over the past 30 years and is expected to continue this positive trend over the next 15 years.

**Conclusion:**

This in-depth analysis based on GBD 2021 provides a fresh perspective on the evolving burden of pediatric urolithiasis in BRICS countries over the last three decades. Our research provides valuable insights for policy makers and health care providers through in-depth analysis and scientific evaluation of the prevalence of pediatric urolithiasis using different statistical models. In addition, BRICS countries should develop targeted prevention strategies for at-risk populations and ensure the availability of effective treatments that are tailored to their national contexts while also reflecting global health trends and evidence.

## 1 Introduction

Pediatric urolithiasis is the formation of stones in the urinary tract in children, mainly in the kidneys, ureters or bladder, and often presents with symptoms such as abdominal pain, haematuria, urinary frequency or urgency (1). The pathogenesis of pediatric urolithiasis is complex and may be closely related to a variety of factors such as genetic factors, metabolic disorders, abnormal urine composition, urinary tract infections and lifestyle (2). In particular, some metabolic disorders, such as hypercalciuria and uric aciduria, increase the risk of the disease in the pediatric population (3). More importantly, pediatric urolithiasis can lead to long-term renal damage, making early diagnosis and intervention crucial (4).

Worldwide, the prevalence of urolithiasis varies considerably from country to country due to many factors including region, dietary habits and environmental factors. Studies have reported the prevalence of urolithiasis to be 1%–5% in Asia, 5%–9% in Europe and 13%–15% in North America (5, 6). Although pediatric urolithiasis is relatively rare compared to adult urolithiasis, its prevalence has been increasing in recent years and accounts for approximately 2%–3% of all urolithiasis cases (6, 7). On the other hand, paediatric urolithiasis can cause abdominal cramps and macroscopic haematuria and is strongly associated with end-stage renal disease in childhood (8–10). Pediatric urolithiasis has attracted widespread public health attention because of the serious threat it poses to children's health and the increasing epidemiological trends.

The BRICS countries, as a group of international organisations and countries representing half of the world's population, are of great importance in the field of public health research (11). The common characteristics of these countries include large population bases, limited public health resources and unique disease burden patterns (12). Therefore, epidemiological assessment of pediatric urolithiasis in the BRICS countries and prediction of future trends will provide an important reference for the development of global public health policies. Over time, the BRICS group has expanded to include new members. However, for the purposes of this study, the BRICS countries referred to are Brazil, Russia, India, China, and South Africa.

The aim of this study is to provide an epidemiological assessment of the disease burden of pediatric urolithiasis in the BRICS countries and to predict future disease trends using data from the GBD Database 2021. Through this analysis, it is expected to provide a scientific basis for the prevention and treatment strategies of pediatric urolithiasis in the BRICS countries as well as globally, and to further promote the improvement and implementation of public health policies.

## 2 Methods

### 2.1 Data resources and definitions

The GBD database was initiated and developed by the World Bank, the World Health Organization and others to estimate the burden of disease and injury on a global scale. The database is based on data from a variety of direct and indirect sources, such as national health surveys, statistical modelling and expert opinion, and is specifically designed to estimate the burden of disease in areas where data are limited. In countries where vital registration systems are in place, mortality and morbidity data are reported directly by the relevant agencies, while in areas where such data are not available, statistical modelling is used to estimate the burden of disease, combining information from household surveys, hospital records and expert opinion.

GBD2021 is the latest version of the database, published on 16 May 2024. This version includes data from 204 countries and territories, covering 371 diseases and injuries and 88 major risk factors. In addition, GBD2021 provides data since 1990, allowing researchers to effectively assess trends and changes in disease burden over time through retrospective analyses.

Pediatric urolithiasis data for the BRICS countries included in this study were obtained from GBD2021, which is freely available on the website (http://ghdx.healthdata.org/gbd-results-tool). It is important to note that the age range of pediatric urolithiasis patients included in this study was limited to 0–14 years due to the inherent format of the GBD data and for better analysis of age subgroups.

### 2.2 Joinpoint regression analysis

Joinpoint regression is a widely utilized statistical technique for examining local trends in disease. It constructs a regression model to identify points of change in time-series data, allowing for the decomposition of an overall trend into distinct subtrends (13–15). This makes the Joinpoint regression model a valuable tool in public health and epidemiological research. The results of the analysis are typically expressed using annual percent change (APC) and average annual percent change (AAPC), with a *p*-value of less than 0.05 indicating statistical significance.

### 2.3 Prediction models

The Autoregressive Integrated Moving Average (ARIMA) model is a widely used method for time series analysis that incorporates three key components: autoregression (AR), integration (I), and moving average (MA) (16–18). These components work together to effectively capture trends and seasonal variations in time series data.

### 2.4 Subgroup analysis

In this study, subgroup analyses were performed to explore the differences in the burden of pediatric urolithiasis in the BRICS countries among different population groups, mainly gender and age groups. Firstly, the burden of disease was divided by gender into two groups, male and female, to highlight potential differences in the burden of pediatric urolithiasis by comparing the burden of disease by gender. Secondly, the age group analysis divided the study population into three age groups, 0–4 years, 5–9 years and 10–14 years, to gain a more detailed understanding of how the burden of disease varies at different ages, as the prevalence and clinical presentation of pediatric urolithiasis may vary with age. Through these subgroup analyses, the study aims to provide data to support the development of more targeted public health strategies and interventions.

### 2.5 Statistical metrics

This study describes the burden of pediatric urolithiasis in BRICS countries between 1990 and 2021 using two statistical parameters: the number of prevalence cases and the age-standardised prevalence rate (ASPR). In addition, the statistical results of this study were expressed using uncertainty intervals (UI) and confidence intervals (CI) to reflect the uncertainty of the estimates. Finally, the above statistical analyses were mainly performed using R software (version 4.2.3) and Joinpoint (version 5.3.0).

## 3 Results

### 3.1 Description of the disease burden

**Figure 1:**
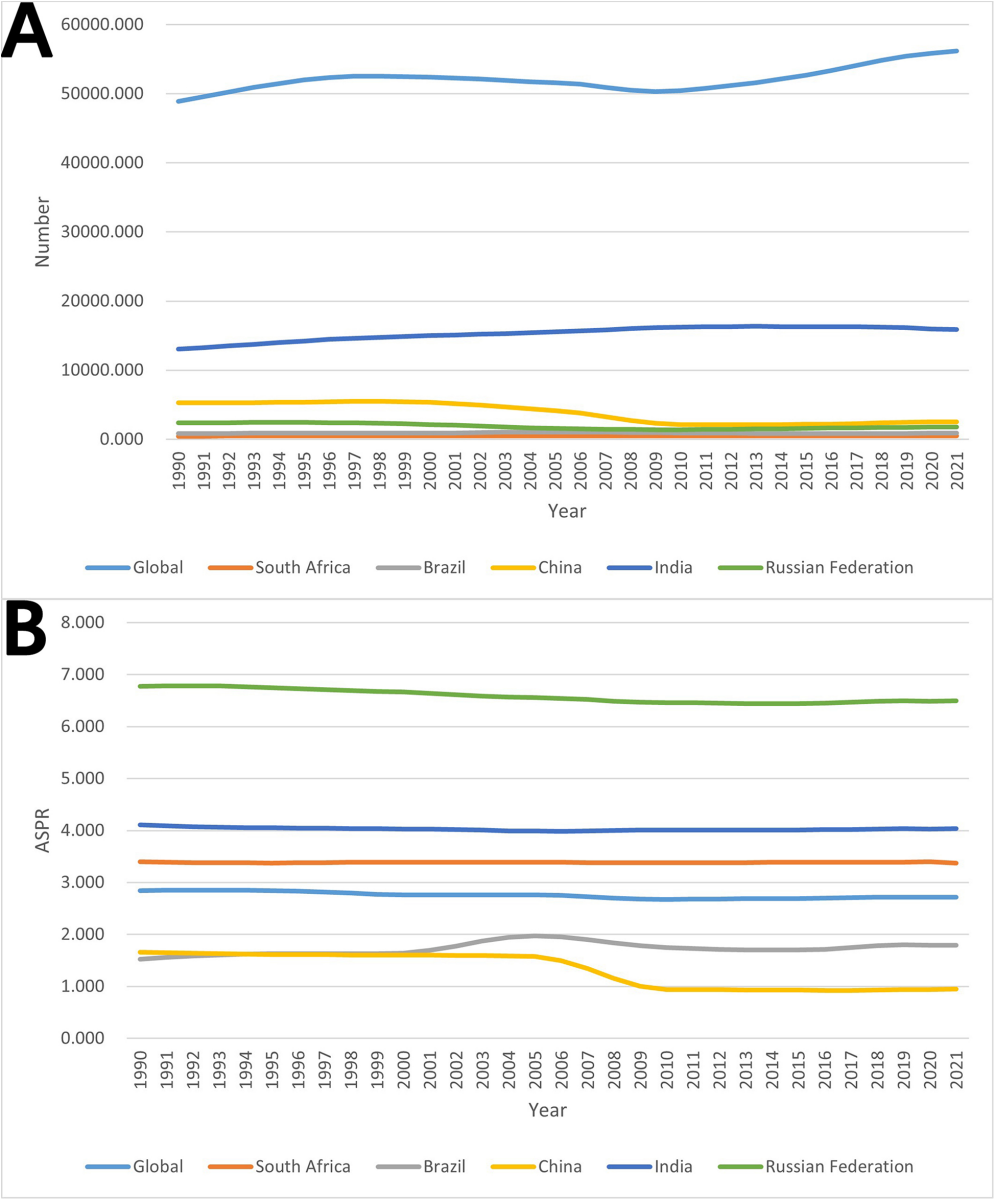
Trends in the burden of pediatric urolithiasis globally and in BRICS countries from 1990 to 2021. **(A)** The number of prevalence cases; **(B)** age-standardized prevalence rate (ASPR).

As shown in Figure 1A; Supplementary Table S1, the global burden of pediatric urolithiasis generally showed an initial increasing, then decreasing and finally increasing trend over the period 1990–2021, with the highest number of prevalence cases being 56,198.398 (95% UI: 32,964.018–86,418.238) in 2021 and the lowest number of prevalence cases being 48,864.936 (95% UI: 27,802.363–76,399.587) in 1990. Among the BRICS countries, India has a much higher prevalence than the other four countries. The disease burden of pediatric urolithiasis in India shows an increasing and then decreasing trend over time. The highest number of cases (i.e., the tipping point) was recorded in 2013 at 16,330.867 (95% UI: 9,492.218–25,041.012). Although the number of cases in 2021 is 15,872.708 (95% UI: 9,207.381–24,317.582), it is still significantly higher than in 1990, when it was 13,072.292 (95% UI: 7,598.348–20,401.749). On the other hand, the burden of pediatric urolithiasis in South Africa is the lowest among the BRICS countries, and the number of cases increases only slightly between 1990 and 2021, with the highest number of cases of 532.239 (95% UI: 305.042–834.817) occurring in 2020 and the lowest number of cases of 457.159 (95% UI: 261.181–716.351) occurring in 1990. The disease burden of pediatric urolithiasis in China increased slowly in two periods, 1990–1998 and 2013–2021, and decreased significantly in 1999–2012. The highest value was 5,495.952 (95% UI: 2,589.344–8,996.477) in 1998; the lowest value was 2,102.646 (95% UI: 1,152.998–3,135.328) in 2012. Disease trends in Russia are similar to those in China, with a rising-falling-rising pattern. The highest level was 2,441.535 in 1994 (95% UI: 1,488.277–3,715.925); the lowest level was 1,405.120 in 2009 (95% UI: 848.364–2,133.810). Brazil shows an increasing and then decreasing trend between 1990 and 2021, with the highest burden in 2005 at 1,061.474 (95% UI: 696.732–1,458.979).

According to Figure 1B; Supplementary Table S2, the global ASPR for pediatric urolithiasis declined slightly between 1990 and 2021, with a level of reaching 2.717 per 100,000 (95% UI: 1.376–4.430) in 2021. Similar to the global trend in ASPR changes, South Africa's ASPR also declines slightly between 1990 and 2021, reaching a level of 3.375 per 100,000 in 2021 (95% UI: 1.695–5.545). At the same time, although Russia's ASPR has shown a slight decline over time, its level is still the highest among the BRICS and well above the global level. Its lowest level was 6.443 per 100,000 in 2014 (95% UI: 3.474–10.368), and its highest level was 6.783 per 100,000 in 1992 (95% UI: 3.657–10.971). The BRICS country with the second highest ASPR after Russia is India. The lowest ASPR in India was 3.988 per 100,000 in 2006 (95% UI: 2.054–6.532); the highest was 4.104 per 100,000 in 1990 (95% UI: 2.121–6.711). On the other hand, China's ASPR is the lowest and has declined significantly over time. Its highest level was 1.658 per 100,000 in 1990 (95% UI: 0.698–2.904) and its lowest level was 0.925 per 100,000 in 2016 (95% UI: 0.421–1.553). Brazil's ASPR is just above China's and is the only BRIC country to show a slight increase over time. Its 1990 level was 1.527 per 100,000 (95% UI: 0.730–2.490) and its 2021 level is 1.795 per 100,000 (95% UI: 0.909–2.914).

### 3.2 Regression analysis

**Figure 2:**
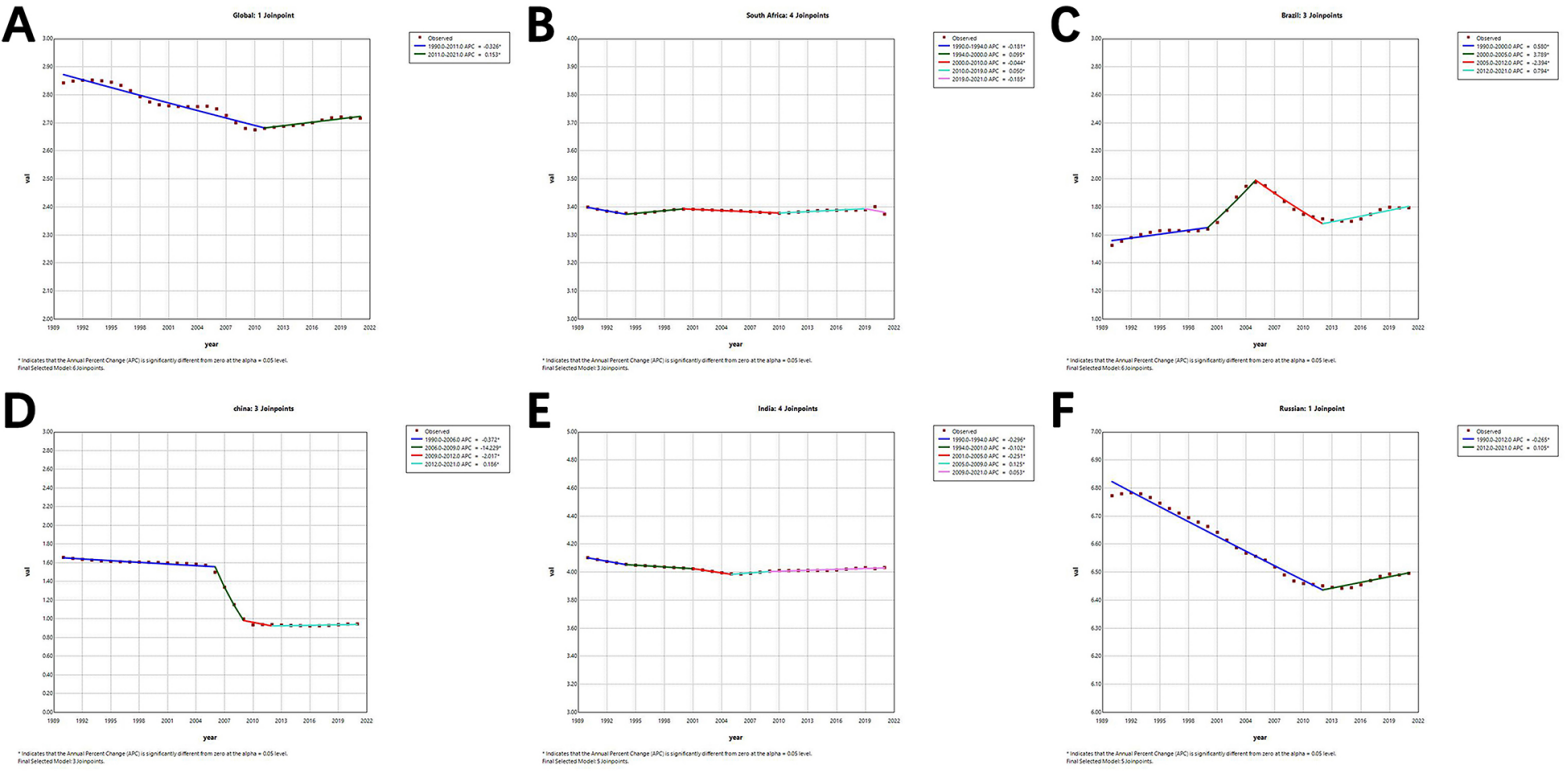
Joinpoint regression analysis in age-standardized prevalence rate (ASPR) for pediatric urolithiasis in global and BRICS countries from 1990 to 2021. **(A)** Global; **(B)** South Africa; **(C)** Brazil; **(D)** China; **(E)** India; **(F)** Russian Federation.

As can be seen from Figure 2; Table 1, the APC and AAPC values of both global and BRICS countries analysed by Joinpoint regression are statistically significant (*p*-value less than 0.05).

**Table 1:** Joinpoint regression analysis: APC and AAPC of ASPR for pediatric urolithiasis in global and BRICS from 1990 to 2021.

	Year	APC/AAPC (%)	95%CI	Test statistic (*t*)	*P*-Value
Global	1990–2011	−0.326	−0.362 to −0.290	−18.481	<0.001
	2011–2021	0.153	0.043 to 0.262	2.864	0.008
	1990–2021	−0.172	−0.213 to −0.131	−8.217	<0.001
South Africa	1990–1994	−0.181	−0.214 to −0.147	−11.368	<0.001
	1994–2000	0.095	0.071–0.118	8.456	<0.001
	2000–2010	−0.044	−0.053 to −0.034	−9.602	<0.001
	2010–2019	0.050	0.039–0.062	9.219	<0.001
	2019–2021	−0.186	−0.291 to −0.080	−3.705	0.002
	1990–2021	−0.017	−0.026 to −0.007	−3.363	0.001
Brazil	1990–2000	0.580	0.338–0.823	4.996	<0.001
	2000–2005	3.789	2.924–4.662	9.235	<0.001
	2005–2012	−2.394	−2.808 to −1.978	−11.850	<0.001
	2012–2021	0.794	0.528–1.062	6.209	<0.001
	1990–2021	0.470	0.282–0.658	4.913	<0.001
China	1990–2006	−0.372	−0.425 to −0.318	−14.485	<0.001
	2006–2009	−14.229	−15.348 to −13.096	−24.316	<0.001
	2009–2012	−2.017	−3.259 to −0.760	−3.323	0.003
	2012–2021	0.186	0.069–0.302	3.309	0.003
	1990–2021	−1.805	−1.973 to −1.636	−20.759	<0.001
India	1990–1994	−0.296	−0.364 to −0.228	−9.142	<0.001
	1994–2001	−0.102	−0.138 to −0.066	−5.890	<0.001
	2001–2005	−0.251	−0.359 to −0.143	−4.889	<0.001
	2005–2009	0.125	0.016–0.234	2.419	0.026
	2009–2021	0.053	0.041–0.066	8.753	<0.001
	1990–2021	−0.057	−0.079 to −0.035	−5.060	<0.001
Russian	1990–2012	−0.265	−0.280 to −0.249	−34.592	<0.001
	2012–2021	0.105	0.045–0.165	3.572	<0.001
	1990–2021	−0.158	−0.177 to −0.138	−15.617	<0.001

AAPC, average annual percent change presented for full period; APC, annual percent change; CI, confidence interval.

Globally, the ASPR for pediatric urolithiasis showed an overall statistically significant downward trend, with an AAPC of −0.172 (95% CI: −0.213 to −0.131). In terms of sub-trends, the ASPR from 1990 to 2011 was improving, with an APC of −0.326 (95% CI: −0.326 to −0.290), while the ASPR from 2011 to 2021 was slightly worsening, with an APC of 0.153 (95% CI: 0.043–0.262). The results of the regression analysis for South Africa are very stable, with an AAPC value of only −0.017 (95% CI: −0.026 to −0.007), although it has a multi-segmented trend, showing a downward trend in 1990–1994, 2000–2010 and 2019–2021, and an upward trend in 1994–2000 and 2010–2019. The results of the regression analyses for India are very similar to those for South Africa. Its overall statistically significant AAPC is only −0.057 (95% CI: −0.079 to −0.035). In terms of sub-trends, India also has five sub-trends and shows a downward trend for 1990–1994, 1994–2001 and 2001–2005 and an upward trend for 2005–2009 and 2009–2021.

Regression analysis of pediatric urolithiasis in China showed a clear trend of improvement overall, with an AAPC of −1.805 (95% CI: −1.973 to −1.636). This is mainly due to the significant decrease between 2006 and 2009, with an APC of −14.229 (95% CI: −15.348 to −13.096). For Russia, the regression analysis shows a slight improvement overall, with an AAPC of −0.158 (95% CI: −0.177 to −0.138). Finally, Brazil is the only BRICS country with a worsening result in the overall regression analysis, with an AAPC of 0.470 (95% CI: 0.282–0.658). Of these, three periods, 1990–2000, 2000–2005 and 2012–2021, show an increasing trend, with the exception of 2005–2012, which shows a decreasing trend.

### 3.3 Predictive analysis

**Figure 3:**
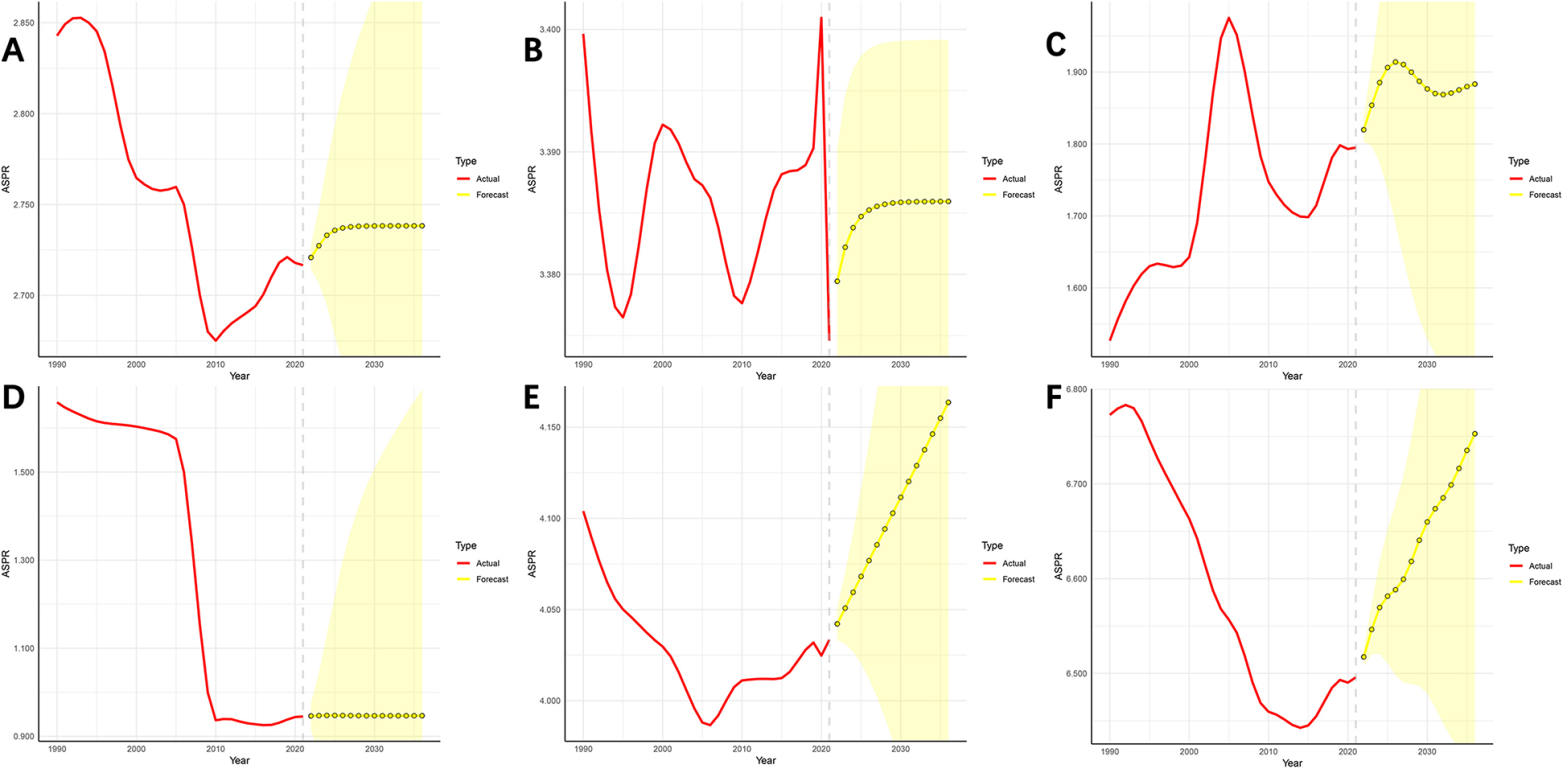
Predictive analysis of pediatric urolithiasis for the next 15 years globally and in the BRICS countries. **(A)** Global; **(B)** South Africa; **(C)** Brazil; **(D)** China; **(E)** India; **(F)** Russian Federation.

Using an ARIMA model, this study predicted the trend of pediatric urolithiasis globally and in the BRICS countries over the next 15 years, and the results can be seen in Figure 3.

First, the global ASPR for pediatric urolithiasis shows a significant upward trend between 2022 and 2025, followed by a gradual stabilisation. South Africa has a similar projected trend to global pediatric urolithiasis, again with a significant increase between 2022 and 2025, followed by a gradual stabilisation. The trend in Brazil over the next 15 years is more variable, showing an increase, a decrease and an overall increase in the burden of disease over the next 15 years. Two BRICS countries, India and Russia, have similar projected trends, with the ARIMA model predicting that the burden of disease in both countries will increase each year between 2022 and 2036. Finally, the predicted trend for pediatric urolithiasis in China is the most specific of the BRICS countries. The ARIMA model predicts that China's ASPR will remain surprisingly stable, maintaining the lowest level among the BRICS countries between 2022 and 2036.

### 3.4 Gender subgroup analysis

Figures, tables and data relating to the analysis of sub-groups by gender can be found in Supplementary Figures S1–S3; Supplementary Tables S3–S5.

#### 3.4.1 Description of the disease burden

Initially, with regard to males vs. females (Supplementary Figure S1; Supplementary Tables S3–S4), the global prevalence of pediatric urolithiasis is much higher in males than in females. The characteristics of pediatric urolithiasis in most of the BRICS countries are the same as globally, with the exception of Russia, which is more specific and shows a higher prevalence of urolithiasis in females than in males. Secondly, the ASPR of pediatric urolithiasis has similar characteristics to the number of prevalent cases, i.e., globally, South Africa, Brazil, China and India have more males than females, and only in Russia is the ASPR higher in females than in males. Finally, the trends in the prevalence and ASPR of pediatric urolithiasis over time for both males and females were similar to those for both sexes as a whole, both globally and in the BRICS countries.

#### 3.4.2 Regression analysis

As shown in Supplementary Figure S2; Supplementary Table S5, the AAPC values for the burden of disease for pediatric urolithiasis in females were statistically significant both globally and in the BRICS countries. On the other hand, among males, only the AAPC for pediatric urolithiasis in South African males was not statistically significant (*p* = 0.906), whereas it was statistically significant in all other BRICS countries and globally.

From a global perspective, ASPR showed an overall decreasing trend for both males and females. China and Russia had similar decreasing trends in ASPR as the world. In addition, South Africa also showed a decreasing trend for female pediatric urolithiasis. Brazil was the only BRIC country to show an overall increasing trend in ASPR for both males and females. Finally, the regression analyses for India were exceptional in that the ASPR for males and females showed overall opposite trends, with a decreasing trend for females and an increasing trend for males.

#### 3.4.3 Predictive analysis

The results of the ARIMA prediction model (Supplementary Figure S3) show that the global ASPR for pediatric urolithiasis in both males and females showed an increasing trend, followed by a gradual stabilisation. The predicted trends in India and Russia are partially similar to the global trends. In India, the ASPR for pediatric urolithiasis showed a gradually increasing trend in females and an increasing stable trend in males. In Russia, the ASPR for pediatric urolithiasis showed an increasing trend in females and a decreasing trend in males. The predicted trends in South Africa and Brazil were also partly similar. In South Africa, the ASPR for pediatric urolithiasis showed a decreasing-increasing-decreasing trend in females and an overall decreasing trend, whereas it showed an increasing-stable trend in males. On the other hand, in Brazil, the ASPR for pediatric urolithiasis in females showed a decreasing-rising-decreasing trend and an overall increasing trend. In contrast, males showed an increasing-decreasing trend and an overall decreasing trend. Finally, China's ASPR remains surprisingly stable for both males and females, and remains the lowest among the BRICS countries between 2022 and 2036.

### 3.5 Age subgroup analysis

See Supplementary Figures S4–S6; Supplementary Tables S6–S8 for figures, tables and data on gender subgroup analyses.

#### 3.5.1 Description of the disease burden

In terms of trends in the burden of disease for pediatric urolithiasis globally and in the five BRICS countries (Supplementary Figure S4; Supplementary Tables S6–S7), India consistently leads the BRICS countries in terms of prevalence, irrespective of age group. It is followed by China, Russia and Brazil, with South Africa consistently having the lowest prevalence. On the other hand, the trend change in ASPR is also very stable, as evidenced by the fact that Russia's ASPR is not only ahead of the other BRICS countries, but also well above the global level. India and South Africa are in second and third place respectively, with China and Brazil always at the bottom. At the same time, when comparing the three age groups (0–4, 5–9 and 10–14), both the global and BRICS countries have the highest prevalence and ASPR in the 10–14 age group and the lowest in the 0–4 age group.

#### 3.5.2 Regression analysis

As shown in Supplementary Figure S5; Supplementary Table S8, the AAPC values for pediatric urolithiasis were statistically significant globally and in the other BRICS countries except Brazil in all three age groups. In Brazil, however, the AAPC for pediatric urolithiasis was only statistically significant in the 5–9 year age group (*p* = 0.475). Globally, China and Russia showed a trend towards decreasing ASPR in all three age groups. South Africa and India showed an overall increasing trend in ASPR in the 0–4 year age group and a decreasing trend in the 5–9 year and 10–14 year age groups. Brazil shows a decreasing trend in the 0–4 age group and an increasing trend in the 10–14 age group.

#### 3.5.3 Predictive analysis

As can be seen from the results of the ARIMA prediction model (Supplementary Figure S6), the ASPR for pediatric urolithiasis worldwide, in Brazil and in Russia showed an increasing trend at 0–4 and 10–14 years and a decreasing trend at 5–9 years. South Africa showed a decreasing trend at 0–4 years and an increasing trend at 5–9 and 10–14 years. Trend projections for changes in disease at 0–4, 5–9 and 10–14 years are consistent between China and India, with China showing a stable trend and India showing an increasing trend.

## 4 Discussion

In this study, various analyses including Joinpoint regression, ARIMA prediction and subgroups were conducted on the burden of disease of pediatric urolithiasis globally and in BRICS countries. Through these analyses, this study is expected to provide a scientific basis for the prevention and treatment strategies of pediatric urolithiasis in BRICS countries as well as globally, and further promote the improvement and implementation of public health policies.

The global burden of pediatric urolithiasis has shown a complex trend over the past decades. The global ASPR for pediatric urolithiasis has shown a decreasing trend, despite an annual increase in the number of cases. This phenomenon may reflect improvements in global public health systems and medical technology, particularly at the level of early diagnosis and treatment (19), which have led to effective case management and treatment despite the increase in new cases. As a result, the global burden of pediatric urolithiasis has been relatively reduced, despite the increase in cases. In addition, all three age groups (0–4 years, 5–9 years and 10–14 years) showed an increasing trend in the number of cases, especially the 10–14 year age group, which is the most significant. The reason for the significant increase in the number of pediatric urolithiasis cases in the 10–14 year age group may be closely related to a number of factors. Firstly, hormonal changes during puberty are a key factor. Fluctuating hormone levels in adolescents can alter the mineral composition of the urine and promote stone formation (20–22). Secondly, increased academic and social pressures may cause some children to experience problems such as irregular diet, lack of exercise, sleep deprivation and poor mental health, which may indirectly affect kidney health and increase the likelihood of urolithiasis (23, 24).

Over the past 30 years, the Chinese government has made remarkable achievements in the prevention and control of pediatric urolithiasis. This is largely due to the high priority given by the government to national health and its continued investment in health care. In 2003 and 2007, the Chinese government launched the government-run New Rural Cooperative Medical Scheme (NRCMS) and the Urban Residents' Basic Health Insurance (URBHI), which provide wider healthcare coverage to the public (25, 26). With improved public health policies and increased medical facilities and technology, pediatric urolithiasis patients have been identified earlier and received timely treatment. In addition, the melamine milk powder incident raised great concern about food safety in society as a whole (27, 28). After the incident, the Chinese government significantly strengthened its food hygiene supervision and gradually established a more scientific and stringent supervision system. At the same time, with the rapid development of Chinese society, public health awareness has increased, and the lifestyle and dietary patterns of children have improved (29). Together, these changes have contributed to a further reduction in the disease burden of pediatric urolithiasis.

The ASPR for pediatric urolithiasis in Russia is not only the highest among the BRICS countries, but also well above the global average. The high ASPR for pediatric urolithiasis in Russia may be closely related to the carnivorous diet and the increased prevalence of obesity and diabetes (30, 31). The underlying mechanism of stone formation in patients with diabetes and obesity is the production of excess uric acid in the urine due to insulin resistance and increased fatty acid production, which in turn leads to the formation of uric acid stones (32). On the other hand, the rapidly increasing alcohol consumption in Russia may also play a role (33), as alcohol and its metabolites can lead to oxidative stress in renal tissue, hypercalciuria and hyperoxaluria, and subsequent stone formation (34).

According to statistical analysis, India has the highest prevalence of pediatric urolithiasis among the BRICS countries. This phenomenon is closely linked to India's explosive population growth and extreme lack of healthcare resources. Studies show that India, with its high birth rate, is expected to overtake China as the world's most populous country in the next decade, with a population of 1.5 billion by around 2025 (35). At the same time, India faces enormous challenges in building a pediatric healthcare system. About a quarter of India's population lacks access to basic healthcare due to difficult economic conditions and a highly unequal distribution of healthcare resources (36). In addition, the number of hospital beds per million people in India is well below the global average set by the World Health Organization (37). The scarcity of health resources has made the Indian government's response to pediatric urolithiasis inadequate.

For pediatric urolithiasis in South Africa, it is easy to see that the trend in the burden of disease in South Africa for ages 0–4 years is increasing, especially after 2005, which is not only contrary to the trends for ages 5–9 and 10–14 years in South Africa, but also to the global and most BRICS trends for ages 0–4 years. This is despite the error associated with sampling variability; after all, the number of urolithiasis cases in the 0–4 year age group is relatively small. However, it cannot be ignored that there is a clear deficiency in the prevention and treatment of pediatric urolithiasis in the 0–4 year age group in South Africa. In addition, the non-significant AAPC and significant APC for the sub-trends that emerged from the Joinpoint regression analysis for pediatric urolithiasis in South African males may be due to the fact that the trends (positive vs. negative) between the different sub-periods cancelled each other out, resulting in a flattening of the overall results. Although the overall AAPC was not significant, the APCs for the different sub-trends were significant, suggesting that there was indeed more variation in changes in the burden of disease over time. Thus, although the long-term trend for pediatric urolithiasis in South African males was not significant, the short-term fluctuations still reflect changes in the burden of disease and the significance of the short-term trend provides valuable information.

Over the last 30 years, although the current burden of pediatric urolithiasis in Brazil is not considered severe, the trend of deterioration has been the fastest among the BRICS countries, and not only that, the trend of deterioration of pediatric urolithiasis in Brazil is expected to continue in the projected analyses. The causes of the worsening of pediatric urolithiasis in Brazil are closely linked to social, economic and environmental factors specific to the country. With the rapid urbanisation of Brazil, more and more families are flocking to the big cities, leading to dramatic changes in lifestyle and eating habits (38). Urban children often consume diets high in salt, sugar and fat and lack physical activity, contributing to the growing problem of obesity, a major risk factor for pediatric urolithiasis (32). At the same time, the Brazilian diet has been influenced by globalisation and industrialised foods, with an increased intake of processed foods and sugary drinks and a decreased intake of fruit and vegetables and fibre-rich foods, an unbalanced diet that contributes to the development of urolithiasis (39). In addition, the inequitable distribution of public health resources in Brazil, especially in poorer areas, makes it difficult to provide medical services that lead to early diagnosis and timely treatment of urolithiasis (40, 41). Despite advances in health education, public awareness of urolithiasis remains limited in parts of Brazil, particularly in poorer areas away from major cities. Finally, the problem is exacerbated by Brazil's hot climate, where high temperatures cause children to lose too much water and concentrate their urine, increasing the risk of urolithiasis (42, 43).

There are some limitations to this study, even though we have chosen to use a variety of models to analyse the data in the latest GBD 2021 database, for the following reasons. First, the data included in the GBD 2021 Database are estimated rather than actual observed data. As a result, the results derived from the modelling of these data may be biased. In addition, there is a lack of more disaggregated analysis in this study to capture the local differences between the regions within the BRICS countries. The healthcare resources of regions within countries differ from their health concerns, and with further analysis this study would provide more valuable information on the management of childhood urolithiasis in BRICS countries and worldwide.

## 5 Conclusion

This in-depth analysis based on GBD2021 provides a fresh perspective on the evolving burden of pediatric urolithiasis in BRICS countries over the last three decades. Our research provides valuable insights for policy makers and health care providers through in-depth analysis and scientific evaluation of the prevalence of pediatric urolithiasis using different statistical models. In addition, BRICS countries should develop targeted prevention strategies for at-risk populations and ensure the availability of effective treatments tailored to their national contexts and global trends.

## Data Availability

The original contributions presented in the study are included in the article/Supplementary Material, further inquiries can be directed to the corresponding author.
