# Supplementary material for: Trend analysis of pediatric urolithiasis prevalence from 1990 to 2021 in the BRICS
Source: Front Pediatr. 2025 Feb 21;13:1551046. doi: 10.3389/fped.2025.1551046 (PMC11885269; doi:10.3389/fped.2025.1551046)
Supplement: Supplementary file 1 [file Table1.docx]

Supplementary Figure

Trend Analysis of Pediatric Urolithiasis Prevalence from 1990 to 2021 in the BRICS


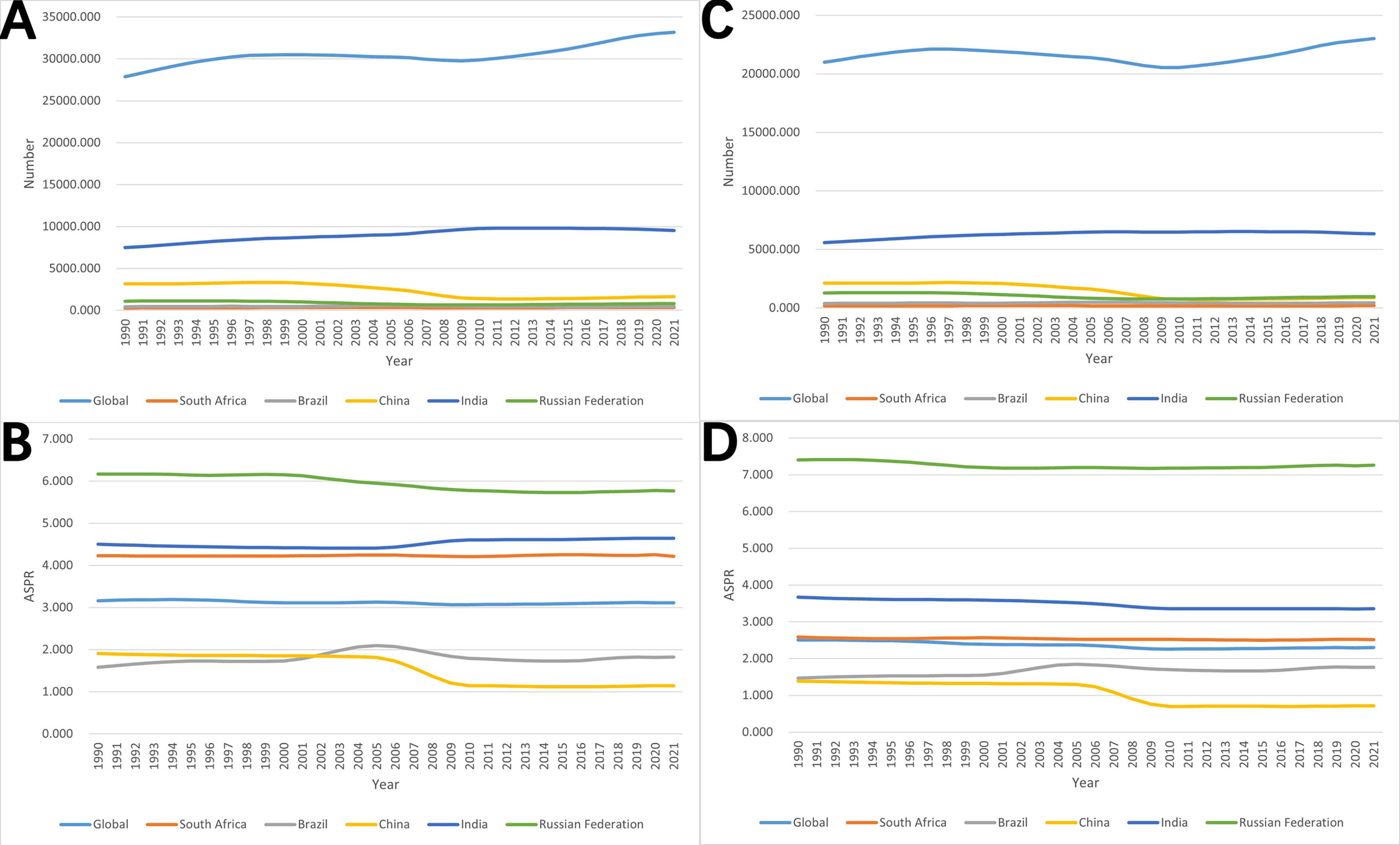


**Figure S1.** Trends in the burden of pediatric urolithiasis globally and in BRICS countries from 1990 to 2021. (A) The number of prevalence cases in male; (B) ASPR in male; (C) The number of prevalence cases in female; (D) ASPR in female.


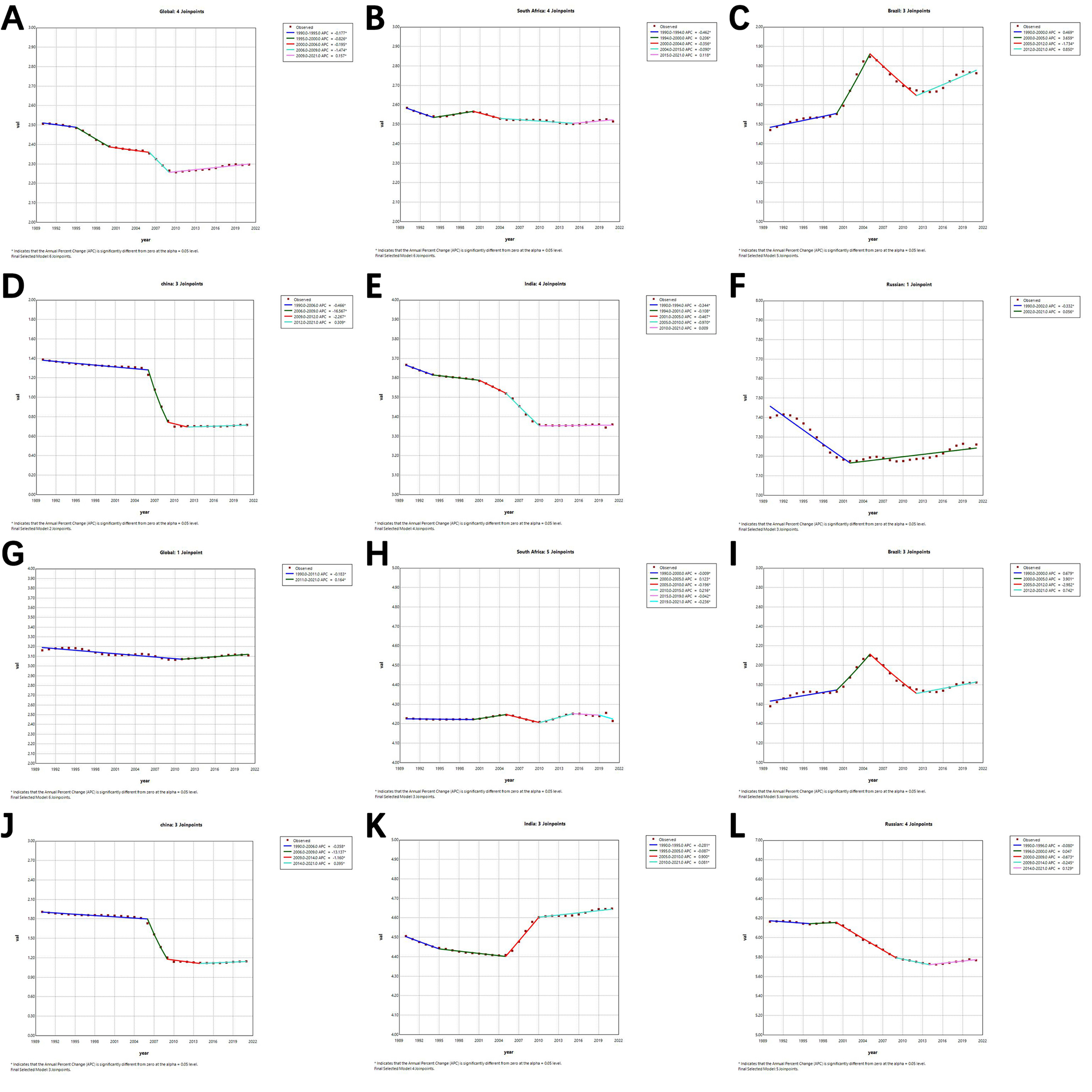


**Figure S2.** Joinpoint regression analysis in ASPR for pediatric urolithiasis in global and BRICS from 1990 to 2021. (A) Global in female; (B) South Africa in female; (C) Brazil in female; (D) China in female; (E) India in female; (F) Russian Federation in female; (G) Global in male; (H) South Africa in male; (I) Brazil in male; (J) China in male; (K) India in male; (L) Russian Federation in male.


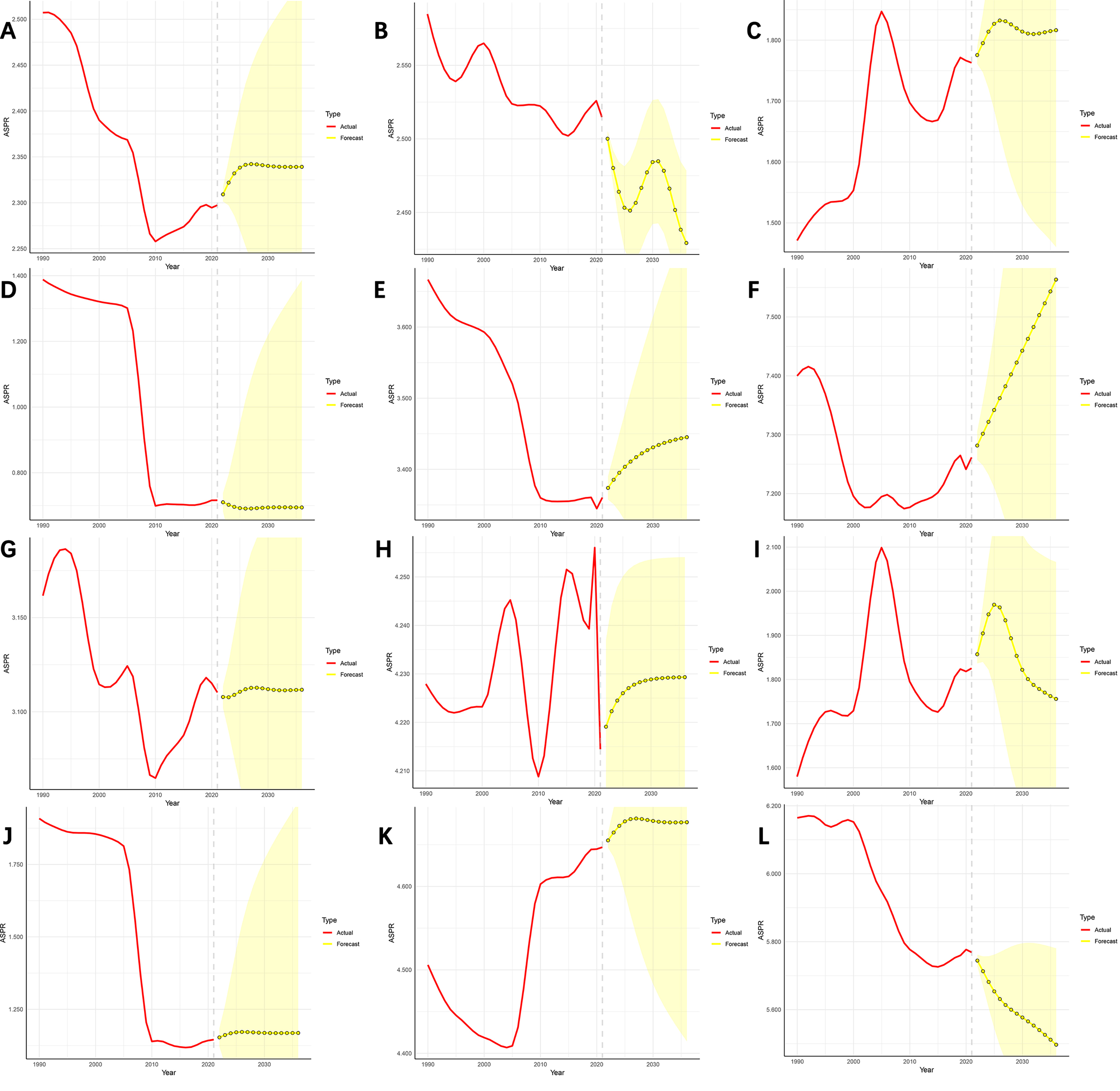


**Figure S3.** Predictive analysis of pediatric urolithiasis for the next 15 years globally and in the BRICS countries. (A) Global in female; (B) South Africa in female; (C) Brazil in female; (D) China in female; (E) India in female; (F) Russian Federation in female; (G) Global in male; (H) South Africa in male; (I) Brazil in male; (J) China in male; (K) India in male; (L) Russian Federation in male.


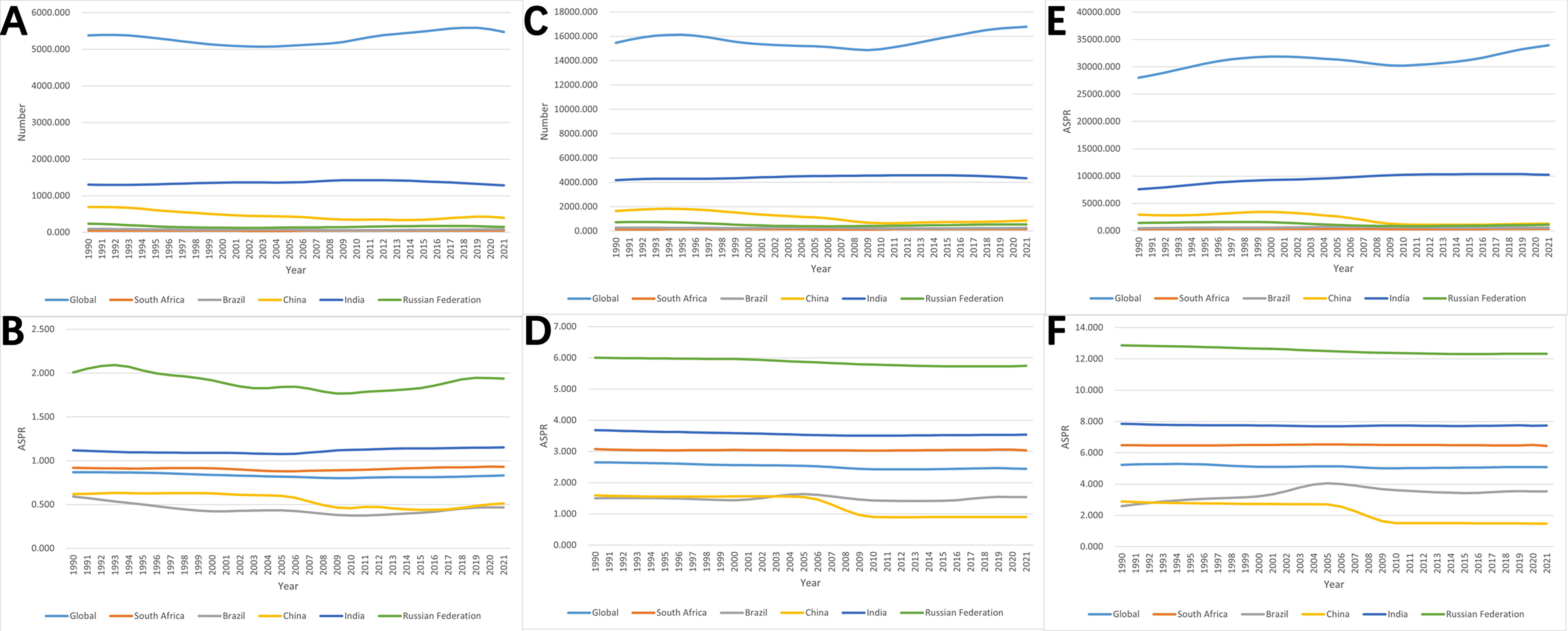


**Figure S4.** Trends in the burden of pediatric urolithiasis globally and in BRICS countries from 1990 to 2021. (A) The number of prevalence cases from 0 to 4 years; (B) ASPR in male from 0 to 4 years; (C) The number of prevalence cases in female from 5 to 9 years; (D) ASPR in female from 5 to 9 years; (E) The number of prevalence cases in female from 10 to 14 years; (F) ASPR in female from 10 to 14 years;


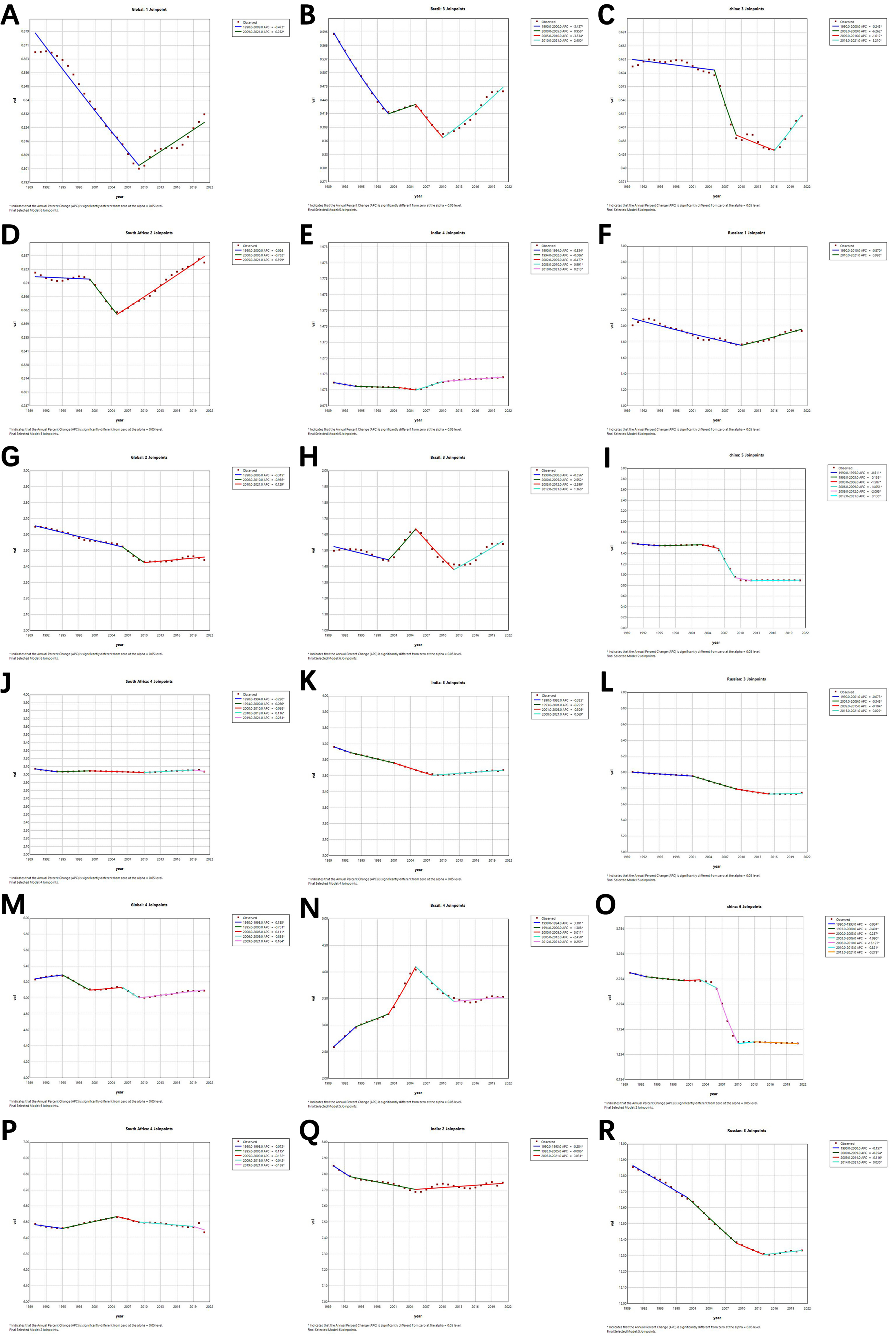


**Figure S5.** Joinpoint regression analysis in ASPR for pediatric urolithiasis in global and BRICS from 1990 to 2021. (A) Global from 0 to 4 years; (B) Brazil from 0 to 4 years; (C) China from 0 to 4 years; (D) South Africa from 0 to 4 years; (E) India from 0 to 4 years; (F) Russian Federation from 0 to 4 years; (G) Global from 5 to 9 years; (H) Brazil from 5 to 9 years; (I) China from 5 to 9 years; (J) South Africa from 5 to 9 years; (K) India from 5 to 9 years; (L) Russian Federation from 5 to 9 years; (M) Global from 10 to 14 years; (N) Brazil from 10 to 14 years; (O) China from 10 to 14 years; (P) South Africa from 10 to 14 years; (Q) India from 10 to 14 years; (R) Russian Federation from 10 to 14 years.


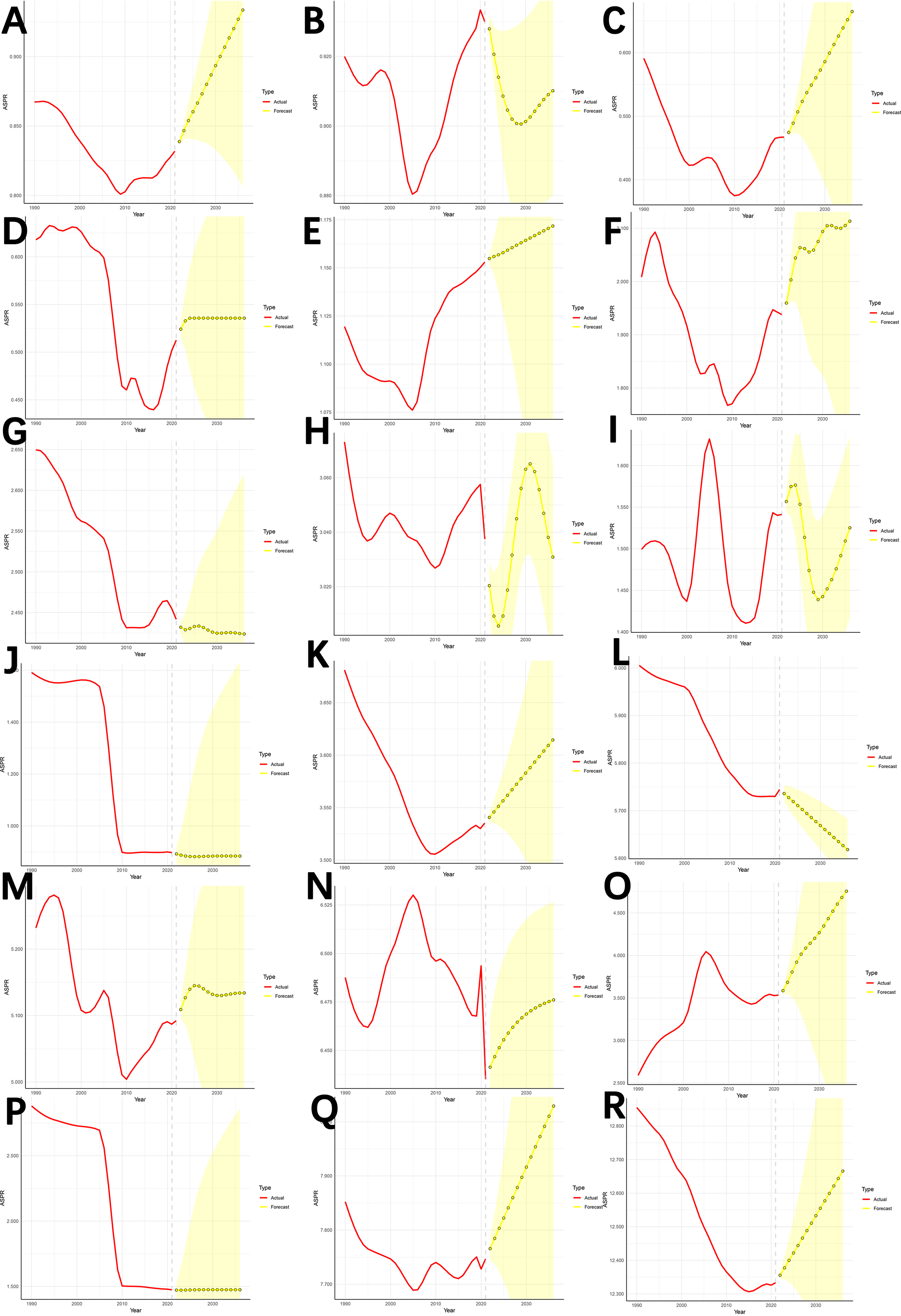


**Figure S6.** Predictive analysis of pediatric urolithiasis for the next 15 years globally and in the BRICS countries. (A) Global from 0 to 4 years; (B) South Africa from 0 to 4 years; (C) Brazil from 0 to 4 years; (D) China from 0 to 4 years; (E) India from 0 to 4 years; (F) Russian Federation from 0 to 4 years; (G) Global from 5 to 9 years; (H) South Africa from 5 to 9 years; (I) Brazil from 5 to 9 years; (J) China from 5 to 9 years; (K) India from 5 to 9 years; (L) Russian Federation from 5 to 9 years; (M) Global from 10 to 14 years; (N) South Africa from 10 to 14 years; (O) Brazil from 10 to 14 years; (P) China from 10 to 14 years; (Q) India from 10 to 14 years; (R) Russian Federation from 10 to 14 years.
